# Reply to Ialongo et al. Vitamin D, SARS-CoV-2 and Causal Associations in Transversal Studies: The Time-Series Analysis to Reveal Potential Confounders. Comment on “Gaudio et al. Vitamin D Levels Are Reduced at the Time of Hospital Admission in Sicilian SARS-CoV-2-Positive Patients. *Int. J. Environ. Res. Public Health* 2021, *18*, 3491”

**DOI:** 10.3390/ijerph18137036

**Published:** 2021-07-01

**Authors:** Agostino Gaudio, Andrea Ruben Murabito, Antonella Agodi, Arturo Montineri, Pietro Castellino, D.O.CoV Research

**Affiliations:** 1 Department of Clinical and Experimental Medicine, University of Catania, 95123 Catania, Italy; pcastell@unict.it; 2 San Marco Hospital, 95121 Catania, Italy; andrearubenmurabito@gmail.com (A.R.M.); a.montineri@libero.it (A.M.); 3 Department of Medical and Surgical Sciences and Advanced Technologies “GF Ingrassia”, University of Catania, 95123 Catania, Italy; agodia@unict.it

We read the comment by Ialongo et al. [1] on our recent article with great interest [2].

We fully agree with our colleagues on the limits that a cross-sectional and transversal study approach can have in analyzing a complex phenomenon that evidently also has its own specific temporal evolution, and all of this was clearly reported in the limitations section of our study [2].

In the last year, numerous articles have focused the spotlight on the possible risk factors associated with SARS-CoV-2 infection, and vitamin D was certainly one of the protagonists among these factors [3].

Vitamin D periodically returns to the forefront of research attention, especially regarding its possible immune-modulating and anti-infective properties. Certainly, there is no lack of evidence in this sense [4], but we believe that vitamin D cannot explain such complex phenomena alone, and low 25-OH vitamin D (25OHD) levels may solely reflect a poor overall health status.

As is well known, the major source of vitamin D is exposure to natural sunlight [5]. In Italy, at least in the first wave of the COVID-19 pandemic, the southern regions were partially spared. This could be partially explained by a recent ecological–statistical study that demonstrated a correlation between COVID-19 deaths and infections with the intensity of solar ultraviolet (UV) radiation at the Earth’s surface, measured in each region by satellite and soil detection [6].

Levels of 25OHD, which represents the marker of vitamin D status, vary during the year due to the different sun exposure levels of the population to UV rays [7]. The patients enrolled in our study obviously belonged to the first wave, and they had low vitamin D values conditioned by the winter–spring period and the national lockdown, which significantly reduced the possibility of the population going outdoors. We also have data relating to patients in the second wave, hospitalized in the period September–November, that we are processing and that show significantly higher vitamin D values compared to the first wave (data not shown).

A further element that should be highlighted is that the approach to diagnosing COVID-19 disease has changed over the months, as Ialongo et al. [1] have reported, because the availability of swabs and our ability to process them changed radically during last summer. This has brought to light cases that were previously not diagnosed, because only clearly symptomatic patients were tested in the previous months.

Therefore, considering these two elements—the variation of vitamin D levels in the population during the year and the different diagnostic approach to SARS-CoV-2 infections—a longitudinal approach could clarify the relationship between vitamin D and COVID-19. In fact, as is well known, longitudinal studies are more likely to suggest cause-and-effect relationships than cross-sectional studies. However, the latter are important because they represent a first step to establishing whether there are links or associations between two or more variables.

Nevertheless, we believe that our study has undeniable strengths, represented by the consecutive enrollment of the studied population, the concomitant dosage of vitamin D levels at hospitalization and the fact that it is the first study conducted in Sicily—the southern region of Italy [2].

Studies with a large population and with a longitudinal design will be required to confirm or rule out an active role of vitamin D in SARS-CoV-2 infection.

## Data Availability

No new data were created or analyzed in this study. Data sharing is not applicable to this article.
